# Short-term L1 arrest duration does not significantly alter organismal phenotypes in synchronized *Caenorhabditis elegans* populations

**DOI:** 10.17912/micropub.biology.002215

**Published:** 2026-07-22

**Authors:** Sourabh Behra, Sambhav Dadsena, Kamesh R. Babu

**Affiliations:** 1 Department of Health Technology, School of Health Sciences and Technology, Energy Acres, UPES, Bidholi, Dehradun-248007, India; 2 Department of Health Sciences, School of Health Sciences and Technology, Energy Acres, UPES, Bidholi, Dehradun-248007, India

## Abstract

Synchronized L1 larvae are commonly maintained under starvation for varying durations prior to experimental use in *Caenorhabditis elegans* research. However, the effects of short-term L1 arrest duration on downstream phenotypes remain unclear. Here, we evaluated developmental growth, reproductive capacity, and locomotor behavior in worms derived from synchronized L1 populations maintained under starvation for 0-6 days before plating. No significant differences were observed in body length, body volume, brood size, embryonic viability, or locomotion among worms derived from different L1 arrest durations. These findings suggest that short-term L1 arrest duration did not significantly influence the organismal phenotypes evaluated in this study.

**Figure 1. Comparison of organismal-level phenotypes across short-term L1 arrest durations in synchronized Caenorhabditis elegans populations:** (A) Schematic representation of the synchronization workflow. Gravid adult worms were subjected to bleaching (0.5% NaOCl and 312.5 mM NaOH) to isolate embryos, which were incubated in M9 buffer at 20 °C with gentle rotation (30 rpm) for ~16 h to obtain synchronized growth-arrested L1 larvae. The synchronized L1 population was maintained under starvation, and aliquots were plated onto PFA-killed OP50-seeded NGM plates following different durations of L1 arrest (0-6 days). Following plating, worms were cultured at 20 °C and developed to the L4 stage within a ~72 h developmental window. (B-C) Body length (µm) and log₂ body volume (µm³) of worms derived from different L1 arrest durations measured at fixed 24-hour intervals following plating (0, 24, 48, 72, 96, and 120 h; total *n* = 500 worms per condition). Under the culture conditions used, these imaging time points corresponded to the predominant L1, L2, L3, L4, young adult, and gravid adult stages, respectively. Two-way ANOVA revealed no significant effect of L1 arrest duration on the body length (P = 0.2481) or body volume (P = 0.3352) growth trajectories across the examined time points. (D-E) Brood size and embryonic viability (%) of worms derived from different L1 arrest durations (total n = 9 worms per condition). Comparable reproductive phenotypes were observed across the tested conditions. (F-G) Body length per second (BLPS; µm/s) and body bends per second (BBPS) of L4-stage worms derived from different L1 arrest durations (total n = 500 worms per condition). No significant differences were observed among the tested groups. Data are presented as mean ± SEM (B-E) or box-and-whisker plots (F-G) derived from three independent experiments.

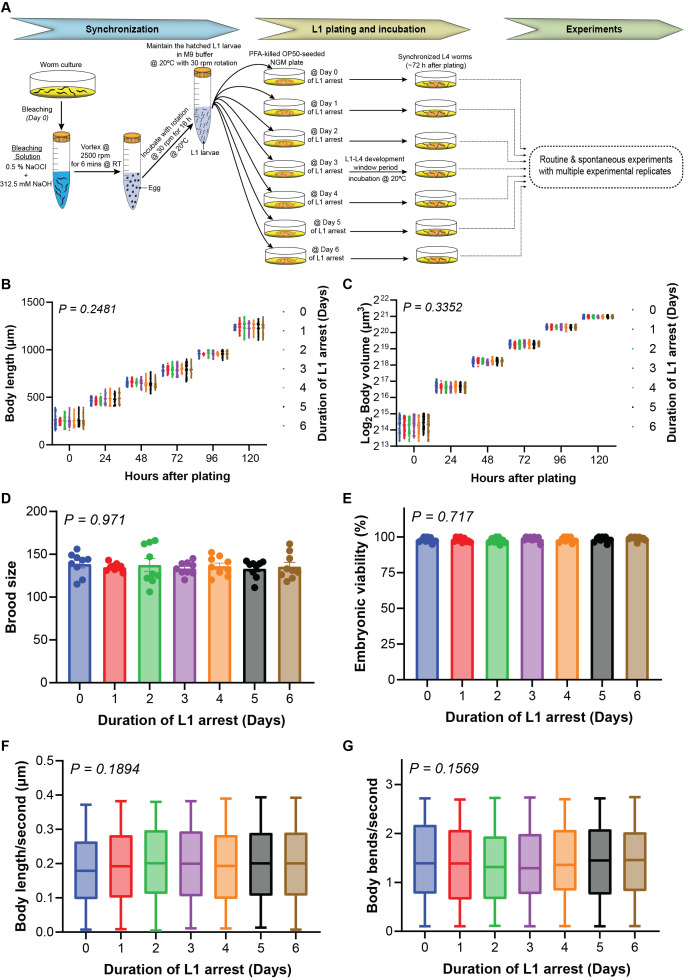



## Description

*Caenorhabditis elegans* ( *C. elegans* ) is a widely used model organism in biological and biomedical research due to its short life cycle, optical transparency, genetic tractability, and suitability for high-throughput experimental studies (Brenner, 1974; Melnikov et al., 2023; Meneely et al., 2019; Packer et al., 2019; Stiernagle, 2006). A fundamental requirement for most *C. elegans* experiments is the generation of synchronous populations, as developmental-stage heterogeneity can substantially influence physiological, molecular, and behavioral readouts (Porta-de-la-Riva et al., 2012; Stiernagle, 2006). Several synchronization methods have been developed, including manual picking (Epstein et al., 1972; Mitchell et al., 1979), filtration-based enrichment (Gandhi et al., 1980; Tilby & Moses, 1975), microfluidics-based sorting (Casadevall i Solvas et al., 2011), and bleaching-induced synchronization (Porta-de-la-Riva et al., 2012; Stiernagle, 2006; Tamez Gonzalez et al., 2024). Among these, alkaline hypochlorite treatment (bleaching) followed by starvation-induced L1 arrest remains the most commonly adopted strategy because of its simplicity, scalability, and ability to generate highly synchronized populations (Fabian & Johnson, 1994; Meneely et al., 2019; Stiernagle, 2006).

During L1 arrest, newly hatched larvae enter a starvation-induced diapause state characterized by developmental quiescence and metabolic adaptation (Baugh, 2013; Johnson et al., 1984; Olmedo et al., 2020). Previous studies have shown that prolonged starvation can influence recovery dynamics, metabolism, fertility, and lifespan (Baugh & Hu, 2020; Jobson et al., 2015; Johnson et al., 1984; Kaplan et al., 2015; Lee et al., 2012; Olmedo et al., 2020; Vogt & Hobert, 2023). In particular, Olmedo et al. (2020) showed that increasing durations of L1 arrest progressively delayed recovery timing following refeeding, including within the first week of starvation (Olmedo et al., 2020). However, while staggered utilization of synchronized L1 populations is commonly practiced informally in many *C. elegans* laboratories, the extent to which variation in short-term L1 arrest duration influences commonly measured downstream organismal phenotypes remains incompletely characterized.

In the present study, we evaluated whether variation in short-term L1 arrest duration (0-6 days of L1 arrest) influences developmental growth, reproductive capacity, or locomotor behavior in synchronized *C. elegans* populations. We further assessed whether synchronized L1 populations maintained under short-term starvation could be practically used to generate synchronized L4 worms across multiple consecutive experimental days from a single synchronization event.

Synchronized L1 larvae were generated by bleaching gravid adults using alkaline hypochlorite treatment, followed by incubation in M9 buffer at 20 °C with gentle rotation for ~16 h to induce starvation-mediated L1 arrest. The synchronized L1 population was subsequently maintained under starvation, and aliquots were plated onto NGM plates seeded with paraformaldehyde (PFA)-killed OP50 at following defined durations of L1 arrest (0-6 days) ( Fig. 1A ). Following plating, worms developed to the L4 stage within approximately 72 h.

To evaluate developmental growth across different L1 arrest durations, worms derived from staggered plating conditions were imaged at fixed 24-hour intervals following plating. Under the culture conditions used, these time points corresponded approximately to the predominant L1, L2, L3, L4, young adult, and gravid adult stages. Body length and body volume were quantified using WorMachine-based image analysis (Hakim et al., 2018). Two-way ANOVA revealed no significant effect of L1 arrest duration on body length (P = 0.2481) or body volume (P = 0.3352) growth trajectories across the examined time points ( Fig. 1B- C). These findings suggest that variation in short-term L1 arrest duration within the tested interval did not significantly influence the measured developmental growth parameters. At first glance, these findings may appear inconsistent with previous reports demonstrating that increasing durations of L1 arrest delay post-starvation developmental progression (Lee et al., 2012; Olmedo et al., 2020). However, the endpoints evaluated differ between these studies. Lee et al. (2012) and Olmedo et al. (2020) primarily quantified developmental timing, including recovery and progression to subsequent larval stages following re-feeding, whereas the present study assessed body length and body volume using the automated WorMachine image analysis platform. In addition, worms were imaged at fixed 24-hour intervals following plating rather than being staged according to the time required to reach specific developmental milestones. Consequently, our measurements reflect morphological growth at predefined imaging time points rather than subtle differences in recovery kinetics or developmental rate. Accordingly, the present study was not designed to quantify recovery time or developmental timing following re-feeding. Thus, while prolonged L1 arrest may delay developmental progression immediately after re-feeding, these differences were not reflected in the organismal growth parameters measured under the conditions of the present study.

Reproductive consistency was assessed by measuring brood size and embryonic viability in worms derived from different L1 arrest durations. Individual synchronized L4 hermaphrodites were transferred daily to fresh plates and reproductive output was quantified across the reproductive period. Comparable brood size (P = 0.971) and embryonic viability (P = 0.717) were observed across all tested conditions ( Fig. 1D- E), indicating that variation in short-term L1 arrest duration did not significantly influence the measured reproductive phenotypes under the present experimental conditions.

Locomotor behavior was analyzed at the L4 stage using video-based tracking (Husson, 2012; Morimoto et al., 2015). Body length per second (BLPS) and body bends per second (BBPS) were quantified using ImageJ and the wrMTrck plugin. No significant differences were observed in BLPS (P = 0.1894) or BBPS (P = 0.1569) among worms derived from different L1 arrest durations ( Fig. 1F- G), suggesting that variation in short-term L1 arrest duration did not significantly influence the measured locomotor parameters.

Collectively, these findings demonstrate that synchronized L1 larvae maintained under starvation for up to 6 days generated broadly comparable organismal-level phenotypes with respect to developmental growth, reproduction, and locomotion under the tested experimental conditions. These observations are consistent with previous studies demonstrating that L1 arrest is a reversible developmental state that preserves organismal viability during short-term nutrient deprivation (Baugh, 2013; Baugh & Hu, 2020; Johnson et al., 1984). However, our findings should be interpreted in the context of previous reports demonstrating that prolonged L1 arrest can alter recovery kinetics, metabolism, stress signaling, and lifespan (Lee et al., 2012; Olmedo et al., 2020). Importantly, the present study primarily evaluated downstream organismal phenotypes at later developmental stages rather than early recovery dynamics immediately following refeeding. Thus, although differences in recovery timing may occur during early post-starvation development, these alterations did not translate into significant differences in the endpoint phenotypes measured here.

From a practical perspective, these findings suggest that synchronized L1 populations maintained under short-term starvation can be used across multiple experimental days to generate synchronized L4 worms while maintaining comparable organismal-level phenotypes across the measured assays. This may reduce the need for repeated synchronization procedures in experiments requiring synchronized populations over consecutive days.

Nevertheless, several limitations should be acknowledged. First, the present study focused primarily on organismal phenotypes and did not investigate molecular or metabolic alterations associated with short-term L1 arrest. Previous studies have shown that starvation can influence stress signaling, metabolic adaptation, recovery dynamics, and lifespan even in the absence of overt developmental defects (Baugh & Hu, 2020; Johnson et al., 1984; Lee et al., 2012). Second, experiments were conducted using PFA-killed OP50 under controlled laboratory conditions, and outcomes may vary with alternative bacterial food sources or environmental conditions. Finally, starvation durations beyond 7 days were not evaluated and may produce different physiological consequences.

In summary, this study characterizes developmental, reproductive, and locomotor phenotypes across varying short-term L1 arrest durations in synchronized *C. elegans* populations. Under the tested experimental conditions, no significant organismal-level differences were observed across the examined L1 arrest durations, supporting the practical use of synchronized L1 populations across multiple experimental days for commonly used phenotypic assays.

## Methods

*C. elegans strain, PFA-killed OP50 and culture conditions*

The culture conditions were followed as previously described (Agrawal & Babu, 2025). Wild-type *C. elegans* ( N2 Bristol) were cultured on 60 mm NGM plates seeded with 30 µL of PFA-killed *E. coli* OP50 . OP50 cultures (500 mL) were grown overnight at 37 °C with shaking (200 rpm) and then treated with 1.25% PFA for 2 h. Cells were washed four times with sterile double-distilled water and resuspended in S-complete buffer at ~5 × 10¹⁰ cells/mL (250 mg/mL). Worms were maintained at 20 °C. Some strains were provided by the CGC, which is funded by NIH Office of Research Infrastructure Programs (P40 OD010440).

*Synchronization of C. elegans population*

Synchronization was performed as previously described (Agrawal & Babu, 2025). Gravid adults from confluent 60 mm NGM plates were collected using 1 mL of M9 buffer and transferred to a 15 mL tube. The volume was adjusted with 13 mL M9 buffer and centrifuged at 1500 rpm for 2 min. After removing the supernatant, the pellet was washed repeatedly with 14 mL M9 buffer until free of bacteria. The pellet was resuspended in 1 mL M9, mixed with 1 mL of 2× bleaching solution, and vortexed at 2500 rpm for 6 min. The reaction was terminated by adding 12 mL M9 buffer, followed by centrifugation at 2000 rpm for 1 min. The pellet was washed three times with M9 buffer, resuspended in 1 mL M9, and incubated at 20 °C with gentle rotation (30 rpm) for approximately 16 h to allow hatching of synchronized L1 larvae. The synchronized L1 population was subsequently maintained in the same buffer under identical conditions at a density of approximately 7,000 larvae/mL (~7 larvae/µL) during the L1 arrest.

*Staggered plating of synchronous starved L1 larvae*

The synchronized L1 suspension was aliquoted and plated onto NGM agar plates seeded with PFA-killed *E. coli* OP50 at following defined durations of L1 arrest (0, 1, 2, 3, 4, 5, and 6 days). Prior to plating, L1 larvae were gently resuspended to ensure a homogeneous distribution. A schematic representation of the staggered plating strategy is provided in Fig. 1A . Following plating, worms were incubated at 20 °C and allowed to develop to the L4 stage. The timing of development was monitored, and worms were collected at the L4 stage for subsequent phenotypic analyses. All plates were prepared under identical environmental and culture conditions to minimize experimental variability.

*Body length and body volume measurement*

Body length and body volume of *C. elegans* were quantified using the WorMachine image analysis platform (Hakim et al., 2018). Worms derived from different L1 arrest durations were imaged at fixed 24-hour intervals following plating. Under the culture conditions used, these imaging time points corresponded approximately to the predominant L1, L2, L3, L4, young adult, and gravid adult stages. Bright-field images were acquired using a Nikon SMZ25 stereomicroscope equipped with a high-resolution monochrome camera (OPTO-EDU 20MPA). All images were captured at 1× zoom under consistent illumination settings to ensure uniform image quality across experimental groups. Captured images were processed using WorMachine implemented in MATLAB (version R2024a), following the standard protocol described in the WorMachine manual (https://github.com/adamhak/WorMachineClient) (Hakim et al., 2018). The software automatically identifies individual worms, segments them from the background, and extracts morphological parameters using skeletonization and feature-extraction algorithms. Body length was calculated based on the worm's skeleton, while additional parameters, including midwidth and thickness, were obtained from the software output. Worm body volume was calculated in Microsoft Excel using an ellipsoidal approximation according to the formula:

$$\mathrm{Volume}=\pi \times \left(\frac{\mathrm{midwidth}}{2}\right)\times \left(\frac{\mathrm{thickness}}{2}\right)\times \mathrm{length.}$$

A minimum of 150 worms per condition were analyzed in each experiment, with a total of 500 worms per condition across three independent experiments. All images were analyzed using identical processing parameters to ensure consistency across samples. Worms that were overlapping, touching, or improperly segmented were excluded from analysis to maintain data accuracy.

*Brood size and embryonic viability scoring*

Brood size and embryonic viability were assessed as previously described (Jaramillo-Lambert & Kwah, 2023). Synchronized L4-stage hermaphrodites obtained from staggered plating following different durations of L1 arrest were individually transferred onto 35 mm NGM plates seeded with PFA-killed *E. coli* OP50 and maintained at 20 °C. Each worm was allowed to lay progeny for a 24 h period, after which the adult worm was transferred daily to a fresh plate for a total of 3 consecutive days. Plates containing laid embryos were incubated at 20 °C for an additional 24 h to allow viable embryos to hatch. Following incubation, the number of live larvae and unhatched embryos on each plate was counted under a stereomicroscope (Nikon SMZ745T). Unhatched embryos after the incubation period were considered non-viable, while hatched larvae were considered viable progeny, consistent with established methods (Jaramillo-Lambert & Kwah, 2023). Brood size was calculated as the total number of progeny (live larvae plus unhatched embryos) produced per worm across all days. Embryonic viability (%) was calculated using the formula:

$$\mathrm{Embryonic viability}(\%)=\left(\frac{\mathrm{Number of live larvae}}{\mathrm{Number of live arvae}+\mathrm{Number of unhatched embryos}}\right)\times 100.$$

For each condition, three individual worms were analyzed per experiment, with a total of nine worms per condition across three independent experiments. All assays were performed under identical experimental conditions to ensure reproducibility.

*Locomotion analysis*

Locomotion of *C. elegans* was analyzed at the L4 stage of worms derived from staggered plating following different durations of L1 arrest using video-based tracking (Husson, 2012; Morimoto et al., 2015). Worms were maintained on 35 mm nematode growth medium (NGM) plates from L1 plating until they reached the L4 stage, after which locomotion recordings were performed. Videos were captured using a Nikon SMZ25 stereomicroscope equipped with a high-resolution monochrome camera (OPTO-EDU 20MPA). Recordings were acquired at 1× zoom, with a frame rate of 28 frames per second (FPS) and a total duration of 60 seconds under consistent illumination conditions. For locomotion analysis, two parameters were quantified: body length per second (BLPS) and body bends per second (BBPS). For BLPS measurements, videos of worms crawling on NGM agar plates were recorded. For BBPS measurements, 200 µL of M9 buffer was added onto the NGM plate to induce swimming behavior before video acquisition. Videos were analyzed using ImageJ (version 1.54f) with the wrMTrck plugin (version 1.04), following the standard protocol recommended in the wrMTrck manual (https://www.phage.dk/plugins/download/wrMTrck.pdf). The plugin performs automated tracking of individual worms and extracts locomotion parameters, including speed normalized to body length (BLPS) and frequency of body bends (BBPS), based on frame-by-frame movement and posture analysis. A minimum of 150 worms per condition were analyzed in each experiment, with a total of 500 worms per condition across three independent experiments. All recordings and analyses were performed under identical experimental conditions to ensure consistency across samples.

*Statistical analysis*

Data are presented as the mean ± standard error of the mean (SEM) from at least three independent experimental replicates. Statistical analyses were performed using GraphPad Prism v.10. A two-way analysis of variance (ANOVA) was performed to compare the body length and body volume growth trajectories among worms subjected to different durations of L1 arrest across the examined imaging time points. A one-way ANOVA test was used to evaluate differences in brood size, embryonic viability and locomotion. The value of *P* < 0.05 was considered significant. Statistical significance indicators are shown in the respective figures.

## Reagents

The bleaching solution, NGM, M9 buffer, S-basal buffer, and S-complete buffer were prepared according to the standard protocols recommended in WormBook (Stiernagle, 2006), and no modifications were made to their compositions.
